# Emergence of Carbapenem-Resistant *Klebsiella pneumoniae* in a Romanian Infectious Diseases Hospital

**DOI:** 10.3390/pathogens14090859

**Published:** 2025-08-29

**Authors:** Dragos Stefan Lazar, Maria Nica, Corina Oprisan, Maricela Vlasie, Ilie-Andrei Condurache, Simin Aysel Florescu, George Sebastian Gherlan

**Affiliations:** 1 Department of Infectious Diseases, “Carol Davila” University of Medicine and Pharmacy, 020021 Bucharest, Romania; dragos.lazar@umfcd.ro (D.S.L.); marce.vlasie@gmail.com (M.V.); simin.florescu@umfcd.ro (S.A.F.); george.gherlan@umfcd.ro (G.S.G.); 2 “Dr Victor Babes” Clinical Hospital of Infectious and Tropical Diseases, 030303 Bucharest, Romania; corina.oprisan@spitalulbabes.ro (C.O.); ilie.condurache@spitalulbabes.ro (I.-A.C.)

**Keywords:** *Klebsiella pneumoniae*, carbapenemases, NDM+OXA48*-like*, antibiotic resistance, colistin, “new” antibiotics

## Abstract

*Klebsiella pneumoniae*, a member of the Enterobacterales Order, often colonises the gut and causes diverse infections, including bloodstream, urinary, and respiratory infections. The rise in carbapenem-resistant sFtrains, especially those producing enzymes like *K. pneumoniae* carbapenemase (KPC), New Delhi metallo-β-lactamase (NDM), Oxacillinase 48 (OXA48), or combinations (NDM+OXA48-*like*), poses a significant threat across Europe, notably in Romania. These strains spread rapidly via mobile genetic elements, complicating treatment. Methods: A retrospective study of multidrug-resistant (MDR) *K. pneumoniae* strains isolated from clinical samples collected at an infectious diseases hospital in Romania. Results: We analysed the evolution of carbapenemases and their combinations from 2010 to 2024, with the rising antibiotic consumption, particularly during the COVID-19 pandemic. The prevalence of carbapenem-resistant *Klebsiella pneumoniae* (CRKP) rose from 4.9% in 2010 to 41.6% in 2024. There was an overall antibiotic use increase, especially colistin (186%) between 2019–2024. Additionally, we examined the dynamics of antibiotic susceptibility that decreased in 2023–2024 and found that susceptibility of NDM+OXA48-*like* isolates to colistin was 16.5% and to cefiderocol 58.5%. Conclusions: The rising prevalence of *K. pneumoniae* strains with complex resistance mechanisms, coupled with a significant reduction in available treatment options, demands a fundamental paradigm shift in the management of these infections.

## 1. Introduction

*Klebsiella pneumoniae* is one of the critical species of the *Enterobacterales* Order. It usually colonises the gastrointestinal tract and is a significant pathogen both in community-acquired and nosocomial infections [1]. It can cause different nosocomial infections: bloodstream, surgical wounds, urinary infections, pneumonia, catheter-associated infections, etc. [2] *K. pneumoniae* is included in the group of ESKAPE pathogens (*Enterococcus faecium*, *Staphylococcus aureus*, *K. pneumoniae*, *Acinetobacter baumannii*, *Pseudomonas aeruginosa*, and *Enterobacter* species), which are responsible for the rise in antimicrobial drug resistance in hospitals [3,4].

Carbapenem antibiotics are considered the most effective antibacterial agents for the treatment of multidrug-resistant bacterial infections. With the widespread use of them, the prevalence of carbapenem-resistant *Enterobacterales* (CRE) has increased rapidly. The difference between CRE and carbapenemase-producing *Enterobacteriaceae* (CPE) is that they were named according to the carbapenem-resistant phenotype and the resistance mechanism. The correct distinction of CRE and CPE and rapid detection of CPE are essential in the treatment and management of clinical infections [5].

Among the four classes of β-lactamases defined by the Ambler classification system, the carbapenemases that confer carbapenem resistance in *Enterobacterales* belong to three of them: Class A (*K. pneumoniae* carbapenemase, KPC), Class B (metallo-β-lactamase, MBL, including New Delhi metallo-β-lactamase, NDM, and the Verona integron- encoded metallo-β-lactamase, VIM) and Class D (oxacillinase β-lactamase-48, OXA-48-*like* carbapenemase) [6].

In the early 2000s, there were only a few clinical records regarding the dissemination of *K. pneumoniae* carbapenem-resistant (CRKP) strains in Romania [7]. According to European surveillance programs of CRE in Europe, from 2004 to 2013, Romania was included in the top six countries with high transmission rates of *K. pneumoniae* resistant to carbapenems. In the same period, the most dominant carbapenemases in Romania and many other European countries were KPC and VIM-type [8,9,10].

Between 2010 and 2012, Szekely et al. made the first report of *bla*NDM-1, *bla*OXA48, and *bla*OXA-181 producing *K. pneumoniae* strains in Romania [11]. During the same years, Gheorghe et al. recorded the geographical distribution of OXA-48 and NDM-1-producing *K. pneumoniae* isolates [12]. From November 2013 to April 2014, Lixandru et al. isolated 65 carbapenem-resistant *K. pneumoniae* (CRKP) strains from eight Romanian hospitals [13]. Most of the isolates carried the *bla*OXA48 resistance gene, followed by the *bla*NDM-1, *bla*KPC-2, and finally the *bla*VIM-1 genes. The resistant genes *bla*OXA48 and *bla*NDM-1 were identified in CRKP strains by many researchers until 2019 [14,15,16,17]. Many studies published between 2021 and 2022 showed the spread of CRKP strains to patients who were hospitalised in the ICU. The majority of CRKP were characterised as OXA48 producers. Increased resistance rates were observed in a variety of antibiotics: fluoroquinolones, carbapenems, and aminoglycosides. Most of the isolates were susceptible to colistin [18,19,20,21].

In 2013, CRKP proportion reached endemic rates in only three out of 27 countries of the European Union: Romania was the third, Italy was second, and Greece ranked first in Europe [22].

In the last 10 years, Romania, Poland, and Denmark have been the countries with the highest rates of NDM-producing *K. pneumoniae* and *Escherichia coli* in Europe [23].

Carbapenem-resistant *K. pneumoniae* emergence poses a severe threat not only to the Balkans but also to all countries in Europe. Greece and Romania have been at an endemic level for the last 20 years. CRKP harbouring *bla*KPC are the most common in these two countries. In the rest of the region, the spread of CRKP with metallo-β-lactamases (MBL), like protagonists, was initiated approximately 10 years later [7].

CPE most often colonises the gastrointestinal tract but can cause urinary tract infection, pneumonia, and bloodstream infections. Antimicrobial resistance genes are included in mobile elements such as plasmids, transposons, and integrons. The importance of these elements lies in their role in the vertical transmission of genes from *Klebsiella pneumoniae* to its descendants, as well as the horizontal transmission of genes from a specific *K. pneumoniae* strain to another [24].

These microorganisms spread very quickly in the hospital environment, through direct contact with another patient who is a carrier of CPE or through the hands of medical staff. Screening of patients from risk groups during admission to the hospital, adherence to hand hygiene procedures by medical staff, and the application of rational antibiotic therapy in healthcare units constitute the primary tools in the fight against the spread of CPE infections [25].

Most CPE strains are entirely resistant to commonly used antibiotics. The treatment of these infections often requires “new” antibiotics or combination antibiotic therapy based on two or three drugs. In infections with CPE strains, there are usually only one or two therapeutic options left for treatment, or the strains are entirely resistant to all known antibiotics. Both laboratories and clinicians are forced to look for combinations of “old” and “new” antibiotics, the combined action of which may provide a chance for therapeutic success [26].

Recently registered new antibiotics such as plazomicin, eravacycline, or cefiderocol may be an effective remedy in the fight against infections caused by CPE strains [27].

In the case of enterobacterial rod-producing metallo-β-lactamases, it is recommended to use cefiderocol or a combination of aztreonam with ceftazidim/avibactam.

The minimum inhibitory concentration (MIC) method is sometimes used in microbiological diagnostics to determine the lowest concentration of an antimicrobial agent that effectively inhibits the growth of a specific microorganism. There are also more specialised diagnostic tools (like perpendicular MIC Test Strip) to assess the interaction of two different antibiotics. This effect may be synergistic, additive, neutral, or antagonistic. Choosing this reciprocal relationship between the two drugs is a crucial therapeutic clue in treating infections caused by carbapenemase-producing *Enterobacterales* [28,29,30,31].

The objectives of our study were:

- to evaluate a dynamic analysis of the evolution of carbapenem resistance in *Klebsiella pneumoniae* strains in the period 2010–2024,
- to assess the potential correlation between the increase in antibiotic consumption during the COVID-19 pandemic and the rise in their incidence;
- to determine the in vitro sensitivity of CPKP isolates collected in the period 2023–2024 to commonly used antibiotics and new molecules, and to highlight the importance of the association of antibiotic resistance phenotypes of CRKP isolates with carbapenemase types.

## 2. Materials and Methods

We conducted a retrospective analysis of multidrug-resistant (MDR) *K. pneumoniae* strains identified from pathological samples (tracheal aspirates, blood, sputum, wounds, urine) collected between 2010 and 2024 at the “Dr. Victor Babeș” Clinical Infectious and Tropical Diseases Hospital in Bucharest (VBH), Romania. The hospital is a tertiary care facility with 490 beds, primarily serving patients with infectious and tropical diseases, and it also includes departments of dermatology and pneumology. The average length of stay is about 6 days. The hospital manages patients with severe or persistent infections or with germs exhibiting multidrug resistance to antibiotics.

The data were collected from the hospital’s information system (InfoWorld, Bucharest, Romania), gathered from clinical departments, the pharmacy, and the microbiology laboratory, and were processed using Microsoft Excel (Version 16.98/2025).

Microbiological evaluation. The study of antimicrobial resistance patterns to compare “old” and “new” antibiotics was performed in the Microbiology Laboratory within the Diagnostic Department of VBH. The analysis was based on clinical samples obtained from the Infectious Diseases, Pneumology, and Intensive Care Units. MDR *Klebsiella pneumoniae* strains were isolated from patient cultures of clinical materials collected for routine microbiological testing, which were then subjected to antimicrobial susceptibility testing, including evaluations of drug combinations, and the selected period for this assessment covered all strains isolated from 1 January 2023 to 31 December 2024. For the period 2010–2023, we used data from the hospital’s microbiology department annual reports. Reported results adhered to CLSI/EUCAST standards applicable at the time.

The study received approval from the Hospital’s Ethics Committee, number 10070/05.06.2025.

Non-repetitive strains of CRKP were tested. When multiple strains exhibiting the same antibiotic resistance phenotype—specifically, the same type of carbapenemase or a combination of carbapenemases—were isolated from different pathological specimens from the same patient, only a single strain per patient was selected for testing.

Identification of the *K. pneumoniae* isolates was performed using an automated system based on mass spectrometry (MALDI-TOF/Matrix-Assisted Laser Desorption/Ionisation—Time of Flight Mass Spectrometry, BRUKER, Bremen, Germany).

Resistance phenotypes (i.e., carbapenem-resistant *Klebsiella pneumoniae*/CRE) were screened using chromogenic media. Brilliance CRE (Oxoid, Basingstoke, UK) is a chromogenic medium containing a modified carbapenem designed to detect bacteria with non-susceptibility to carbapenems.

Antimicrobial susceptibility testing was conducted using the standardised Kirby–Bauer disk diffusion method (30 µg/disc, Oxoid) for the following antibiotics: ampicillin, amoxicillin/clavulanic acid, piperacillin/tazobactam, cefuroxime, ceftriaxone, ceftazidime, cefepime, imipenem, meropenem, amikacin, gentamicin, levofloxacin, ciprofloxacin, trimethoprim/sulfamethoxazole, ceftazidime/avibactam, ceftolozane/tazobactam, imipenem/relebactam, and meropenem/vaborbactam (CLSI between 2010–2019 and EUCAST between 2019–2024).

MICRONAUT-S test plates (BRUKER, Bremen, Germany) are in vitro diagnostic devices used for: (1) quantitative susceptibility testing of clinically relevant, fast-growing Gram-negative bacteria (Enterobacterales and non-fermenters), and (2) qualitative phenotypic detection and differentiation of relevant carbapenemases. Susceptibility testing against antibacterial agents was performed using Mueller–Hinton Broth, cation-adjusted (CAMHB). Antibiotic and β-lactamase inhibitor combination susceptibilities were determined by measuring minimum inhibitory concentrations (MICs) according to EUCAST guidelines for 2023/2024. Results were read using the plate reader (TECAN, Salzburg, Austria) and analysed with MICRONAUT6 software. Cefiderocol susceptibility was assessed via broth microdilution using individual MICRONAUT strips (See Appendix A).

Phenotypic detection of carbapenemases was performed with the RESIST-5 O.O.K.N.V. immunochromatography test (CorisBioConcept, Gembloux, Belgium). This lateral flow assay detects and identifies carbapenemases directly from bacterial colonies using membrane technology with colloidal gold nanoparticles. Quality control was conducted using the reference strain *E. coli* ATCC 25922.

Molecular techniques are considered the gold standard for the identification of carbapenemase genes [32]. In our study, a PCR-based method in a cartridge format designed to detect carbapenemase-producing Enterobacteriaceae (CPE) directly from rectal swabs or pure *K. pneumoniae* cultures was used. This method is run on the GeneXpert^®^ platform (Cepheid, Sunnyvale, CA, USA) and offers high sensitivity (96.6%) and specificity (98.6%) within a short turnaround time (32–48 min). The Xpert Carba-R assay, performed on the GeneXpert^®^ system, is a qualitative in vitro diagnostic test designed to detect and differentiate the *bla*KPC, *bla*NDM, *bla*VIM, *bla*OXA-48, and *bla*IMP gene sequences associated with carbapenem non-susceptibility. The test utilises automated real-time PCR and is intended as an aid in infection control for the detection of carbapenem-non-susceptible bacteria colonising patients in healthcare settings.

The FilmArray Pneumonia plus panel testing was performed according to the manufacturer’s instructions. In this system, automated nucleic acid extraction, multiplex PCR, and post-amplification analysis are conducted within the FilmArray instrument. The panel detects antibiotic resistance genes like *mec*A/*me*C and those codifying the enzymes CTX-M, IMP, KPC, NDM and OXA-48-*like*. The generated report documents the identified organisms and resistance genes [33].

The in vitro efficacy of two antibiotic combinations—ceftazidime/avibactam and aztreonam—was evaluated for *K. pneumoniae* isolates using the E-test cross method [34]. The ceftazidime/avibactam (CAZ/AVI) E-test (BioMérieux, Marcy-l’Etoile, Craponne, France), containing CAZ (0.016–256 µg/mL) and AVI (4 µg/mL), was used alongside aztreonam to assess potential synergy. MIC values for ceftazidime/avibactam and aztreonam were determined both individually and in combination via MIC test strips.

Mueller–Hinton agar plates were inoculated with a 0.5 McFarland standard suspension, and E-test strips were placed at a 90° angle crossing at the MIC points of the individual agents. After incubation for 18 h, the zones of inhibition were read, and the fractional inhibitory concentration index (FIC index) was calculated and interpreted. The FIC index (Σ FIC) was determined using the following formula:

Σ FIC = FIC (A) + FIC (B).(1)

FIC (A) = MIC of drug A in combination with drug B/MIC of drug A alone.

FIC (B) = MIC of drug B in combination with drug A/MIC of drug B alone.

The Σ FIC values determine the effect of the tested combination as follows: ≤0.5 (synergy); >0.5 to ≤1.0 (additively); >1.0 to <4.0 (indifference); and ≥4.0 (antagonism) [35].

Multidrug resistance (MDR) was defined according to non-susceptibility to at least one agent in ≥3 antibiotic classes. Extensive drug resistance (XDR) was found when isolates were susceptible to a maximum of two agents in the same or different antimicrobial categories [36].

Patients from whom carbapenemase-producing *K. pneumoniae* (CPKP) strains isolated were investigated upon admission for colonisation with multidrug-resistant bacterial species through microbiological screening swabs (nasopharyngeal, rectal, inguinal, and axillary). These patients met the clinical and epidemiological criteria for microbiological screening (e.g., transfer from another hospital or nursing home, hospitalisation within the last three months, long-term broad-spectrum antibiotic therapy, etc.).

We monitored patient colonisation at the time of admission with MDR species from the ESKAPE group, particularly *K. pneumoniae*, using chromogenic culture media. Brilliance™ CRE Agar (ThermoFisher, Waltham, MA, USA) provides presumptive chromogenic identification of carbapenem-resistant *E. coli* and bacteria from the *Klebsiella*, *Enterobacter*, *Serratia*, and *Citrobacter* (KESC) groups directly from clinical samples within 18 h.

## 3. Results

Starting in 2010, when we first isolated carbapenem-resistant *K. pneumoniae* (CRKP) strains, the focus was set on analysing the evolving dynamics of antibiotic resistance among these multidrug-resistant (MDR) isolates within our hospital. In 2010, the incidence of CRKP strains was 4.9%. Between 2011 and 2018, a rising trend was observed in the isolation of these strains, increasing from 7.1% in 2011 to 27.6% in 2017.

During the COVID-19 pandemic years, significant increase in the isolation rate of CRKP strains was noted: 37.4% in 2020, 40.0% in 2021, and 34.8% in 2022. This increase was associated with bacterial co-infections related to COVID-19/SARS-CoV-2 infection, extended hospital stays, a higher number of moderate and severe clinical cases, and increased antibiotic use, including broad-spectrum β-lactams/third-generation cephalosporins, carbapenems, fluoroquinolones, macrolides, etc.

**Figure 1:**
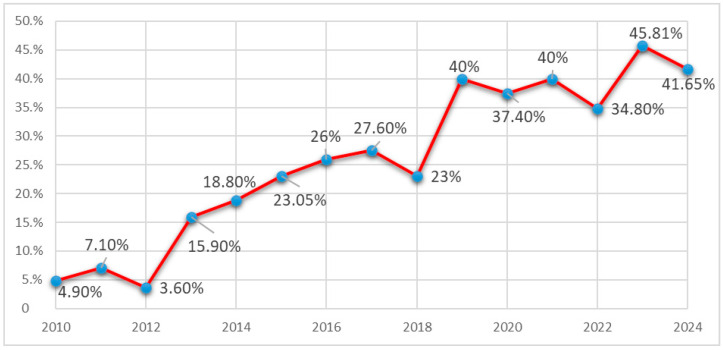
Evolution of CRKP Strain Isolation Rates from 2010 to 2024.

In the post-pandemic years, the incidence of CRKP isolates remained above 40%, with rates of 45.81% in 2023 and 41.65% in 2024 (see Figure 1).

From 2018 to 2024, our hospital systematically monitored the evolution of carbapenemase types in *K. pneumoniae* strains through comprehensive phenotypic and genotypic analyses. This approach allowed us to assess the dynamic changes in the distribution and prevalence of different carbapenemase enzymes over the specified period. Until 2022, not all studied CRKP strains could be classified. The presence of NDM+OXA48-*like* was first reported in 2021 and quickly gained prevalence over other carbapenemase types or their combinations.

This dynamic is reflected in Table 1, which summarises the evolving distribution and prevalence of different carbapenemase types over the years.

**Figure 2:**
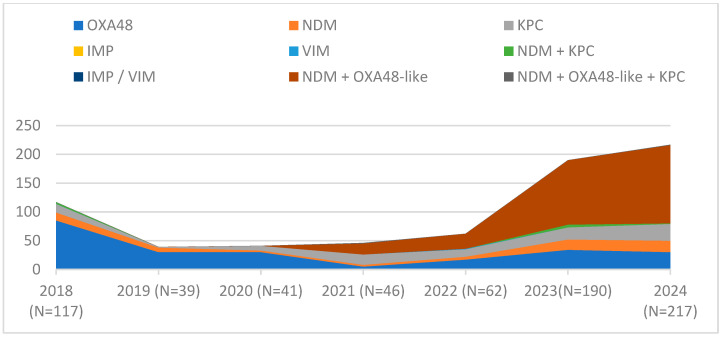
CRKP strains identified at VBH (2018–2024).

The presence of NDM+OXA48-*like* was first reported in 2021, during the COVID-19 pandemic, when all hospitalised patients that year had SARS-CoV-2 infection with multiple complications, including bacterial infections. This combination of carbapenemases quickly increased in prevalence, becoming the dominant profile starting from 2023. These changes in the structure of carbapenemases are illustrated in Figure 2.

Following the emergence of the NDM+OXA48-*like* combination, it gradually occupied this “ecological niche”. Between 2019 and 2022, the number of strains identified as CRKP remained relatively stable. However, starting in 2022, the number of carbapenem-resistant strains showed a consistent increase, almost exclusively driven by the NDM+OXA48-*like* combination.

Furthermore, antibiotic consumption was analysed between 2019 and 2024 to investigate any potential temporal link with the rising number of identified CRKP strains. To achieve this, we retrieved data on antibiotic usage from the VBH informatics system, expressed in therapeutic units (TU), and compiled quarterly reports for the specified years. We had to exclude data on antibiotic consumption from our study in 2018, as they were not fully available.

Throughout the studied period, there was a general moderate increase in total antibiotic use within the hospital. Notably, a significant peak occurred during the winter of 2020–2021, coinciding with a high number of patients infected with SARS-CoV-2 presenting moderate to severe illness. Another smaller rise in antibiotic consumption was observed in the latter half of 2021, which was correlated with cases involving the *Delta* variant of the virus.

While increases across multiple antibiotic classes were recorded during these times, the predominant usage was of injectable beta-lactams, particularly meropenem and ceftriaxone.

Starting in 2022, antibiotic consumption continued to rise, reaching a plateau in the second half of 2023 that persisted into 2024. Throughout this period, the use of injectable beta-lactams remained relatively stable; however, there was a noted increase in the use of other antibiotics, especially colistin, which is employed in treating infections caused by multidrug-resistant Gram-negative bacteria.

**Figure 3:**
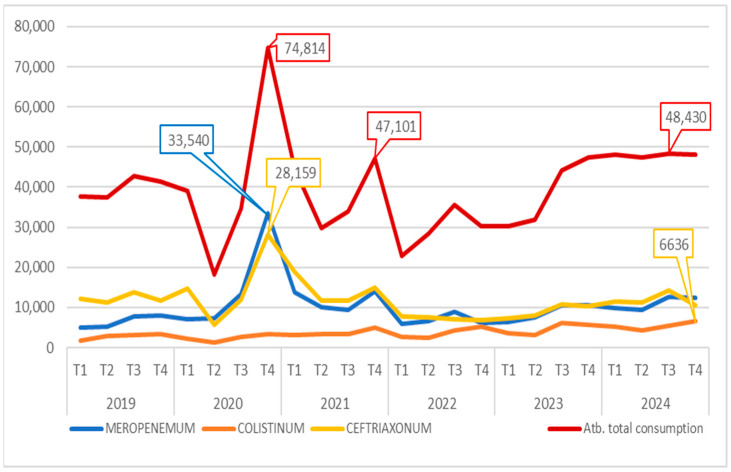
Evolution of antibiotic usage (TU) recorded in VBH (2019–2024).

Analysis of antibiotic consumption revealed a steady increase in the use of colistin over the studied period, from 11,787 TU in 2019 to 21,870 TU in 2024. Concurrently, there was a notable rise in the use of newer antibiotics, correlating with their availability for patient treatment. These findings support the concept of an increasing prevalence of infections caused by Gram-negative bacteria with multiple resistance mechanisms. These data are illustrated in Figure 3.

**Figure 4:**
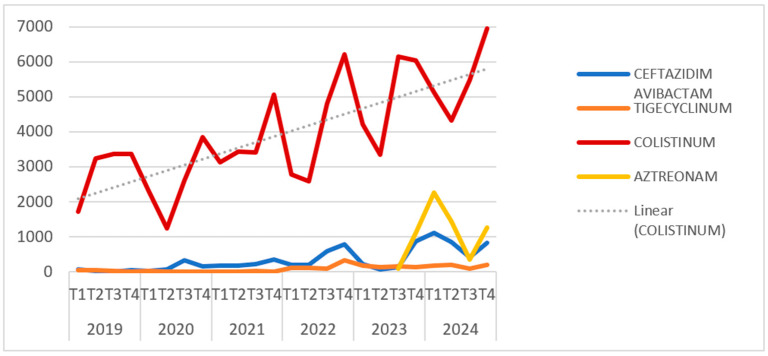
Dynamics of colistin usage compared to “new” antibiotics (TU) in VBH (2019–2024).

Correlating Figure 2, Figure 3 and Figure 4, can be observed that the increase in the prevalence of CRKP NDM+OXA48-*like* occurred approximately one year after the peaks in beta-lactam consumption recorded during the pandemic period, alongside a steady rise in colistin use throughout the studied period (2019–2024).

In the second phase of the study, a total of 439 clinical specimens were analysed. 208 specimens collected in 2023 and 231 in 2024—from hospitalised patients at VBH between January 2023 and December 2024. These samples were obtained from the departments of Infectious Diseases, Pulmonology, Dermatology, and Intensive Care.

In 2023, various types of biological samples were examined, leading to the isolation of 190 unique CRKP strains. The distribution of these isolates by specimen type was as follows: urine (60.3%), purulent secretions (14.2%), lower respiratory tract secretions (13.7%)—including bronchial aspirates (10.4%) and sputum (3.3%)—blood (9%), and other specimen types (2.8%).

**Figure 5:**
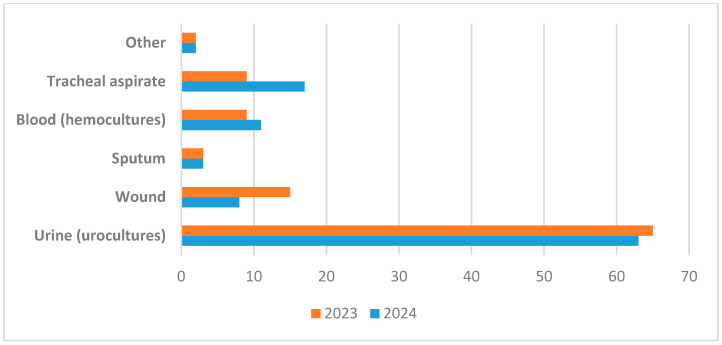
Distribution of CRKP strains by sample type detected during 2023–2024. Numbers on x axis represent number of respective samples.

In 2024, microbiological testing was performed on 231 specimens, resulting in the isolation of 217 distinct CRKP strains. The distribution by specimen type was: urine (59.0%), lower respiratory secretions (20.3%)—including bronchial aspirates (17.1%) and sputum (3.2%)—purulent secretions (7.8%), blood (hemocultures) (10.6%), and other specimen types (2.3%). These findings are summarised in Figure 5.

In 2023, 406 non-repetitive strains of *K. pneumoniae* were tested, isolated from various pathological specimens, collected for diagnostic purposes, from hospitalised patients. Resistance testing (broth microdilution method (BMD)/Micronaut/Bruker) to the most frequently used antibiotics of *K. pneumoniae* strains revealed: 67% resistance to amoxicillin–clavulanic acid, 66.7% resistance to ceftriaxone, 50% resistance to gentamicin, and 66.7% resistance to ciprofloxacin. The resistance of *K. pneumoniae* strains to third-generation cephalosporins (CFIII) was 66.7% for ceftriaxone and 64% for ceftazidime, the difference being due to the isolation of extended-spectrum β-lactamase (ESBL) producing strains of cefotaximase/CTX-M-*like* type. The percentage of resistance to CFIII is the result of combining resistance through ESBL production (20.9%) and resistance through carbapenemase production (45.8%). Lower resistance values were recorded for piperacillin/tazobactam (59.1%) and amikacin (43.3%). Resistance to meropenem was 44.6% (Table 2).

In 2024, a total of 521 non-repetitive strains of *K. pneumoniae* from various types of specimens were isolated. Resistance to the most commonly used antibiotics, representatives of standard treatment classes, was as follows: amoxicillin/clavulanic acid 63%, ceftriaxone 63.8%, gentamicin 47.5%, ciprofloxacin 62.5%, and meropenem 41.4% (Table 2). Out of the *K. pneumoniae* strains, 117 (22.4%) were identified as producers of extended-spectrum β-lactamases (ESBL), with 2.6% specifically producing cefotaximase-type enzymes (ESBL/CTX-M-*like*). The resistance to third-generation cephalosporins (CFIII) is the combined result of resistance caused by both ESBL and carbapenemase production (CRKP = 217/41.4%).

Although the number of isolated strains increased significantly from 406 to 521, the data from Table 2 show that resistance slightly decreased for most antibiotics between 2023 and 2024, except Piperacillin–tazobactam.

In 2023, carbapenem resistance was attributed to 190 CRKP strains: 21 producing KPC, 35 producing OXA48, 18 producing NDM, 110 producing both NDM and OXA48, and 5 producing both NDM and KPC. Notably, the most frequent carbapenemase combination identified among these strains was OXA48 plus NDM, accounting for 58% of all tested CPKP strains.

In 2024, among the 217 CRKP strains isolated, phenotypic and genotypic methods identified the following carbapenemase types: 20 KPC, 30 OXA48, 29 NDM, 136 NDM+OXA48-*like*, and 1 NDM + KPC strain (Table 3). In one case, a triple combination of carbapenemases NDM+OXA48-*like* + KPC was also identified.

Table 3 reveals that the NDM+OXA48*-like* combination was the most prevalent carbapenemase type in both 2023 and 2024, with a noticeable increase in both proportion and absolute numbers over this period. A similar, though less pronounced, upward trend was observed for strains producing NDM alone. In contrast, the prevalence of OXA48 and KPC carbapenemases showed a declining trend. Throughout the two years, carbapenemase enzymes of the VIM class were absent. Of particular concern is the emergence in 2024 of strains harbouring the triple combination NDM + KPC + OXA48, which may pose significant challenges in the future.

In 2023, seven out of 35 *K. pneumoniae* OXA48-producing strains exhibited susceptibility (S: susceptibility at standard doses; I: susceptibility at increased exposure) to meropenem, imipenem, and ceftriaxone or cefotaxime. All of these strains were fully susceptible to amikacin and ceftazidime/avibactam; three out of seven were susceptible to gentamicin; one out of seven to trimethoprim/sulfamethoxazole; and two out of six to colistin. Additionally, six out of seven strains were resistant to ertapenem. These findings are presented in Table 4.

In 2024, seventeen out of 30 OXA48 carbapenemase-producing strains exhibited susceptibility, either at standard doses or intermediate susceptibility with increased exposure according to EUCAST, to various antibiotic classes, including carbapenems and third-generation cephalosporins (CFIII). Specifically, 8 of 30 were susceptible to meropenem, 7 of 30 to ceftriaxone, 5 of 30 to gentamicin, 10 of 30 to amikacin, 3 of 30 to trimethoprim–sulfamethoxazole, 12 of 30 to colistin, and 14 of 30 to cefiderocol. All 30 OXA48-producing CPKp strains were fully susceptible to the combination of ceftazidime-avibactam (Table 5).

In the years 2023 and 2024, we analysed the origin of *K. pneumoniae* strains producing various types of carbapenemases by having the attending physician complete a Clinical and Epidemiological Data Form. This form emphasised patient transfers from other hospitals, nursing homes, or other communal settings, hospital admissions within the previous 3 months, the type of antibiotics received during this period, or whether the patient was undergoing antibiotic treatment at the time of admission, including which antibiotics were being used.

Patients who met these criteria upon admission underwent microbiological screening to detect colonisation by multidrug-resistant (MDR) organisms, including *K. pneumoniae* producing ESBL or carbapenemases (CRKP) from the ESKAPE group.

A total of 124 patients met the clinical and epidemiological criteria and were screened for nasopharyngeal and rectal carriage of CRKP. Among these, 32 patients were found to be colonised with CRKP upon admission: 3 with KPC, 5 with NDM, 8 with OXA48, and 16 with the combination NDM+OXA48-*like*.

During the same period, 177 patients was found with bacterial infections at various sites caused by CRKP, distributed as follows: 17 (10%) with KPC, 28 (15.8%) with NDM, 31 (17.5%) with OXA48, 99 (55.9%) with NDM+OXA48-*like*, and 2 (1.1%) with NDM + KPC.

Both colonised and infected patients at admission predominantly originated from other multidisciplinary hospitals, particularly from surgical departments such as urology, general surgery, and neurosurgery.

CRKP strains were isolated from inpatients at our tertiary care hospital, with their distribution reflecting the profiles of various clinical departments. Given the hospital’s primary focus on infectious and tropical diseases, the isolates were categorised by department as follows: Infectious Diseases (246 strains), Pulmonology (21 strains), Intensive Care Unit (74 strains), and Dermatology, along with outpatient clinics (66 strains). Notably, *K. pneumoniae* strains producing both OXA48 and NDM carbapenemases were predominantly isolated from the Infectious Diseases department (144 strains), the Intensive Care Unit (56 strains), Pulmonology (8 strains), and other hospital units, including outpatients (40 strains).

Antibiotic susceptibility testing of CRKP strains using broth microdilution (Micronaut/Bruker) yielded the following results:

In 2023, isolates from the OXA48/KPC group demonstrated 48% susceptibility to colistin and 75.6% to cefiderocol. All strains in this group remained susceptible to the ceftazidime/avibactam combination. NDM-producing strains showed 50% susceptibility to colistin and 58.3% to cefiderocol. Within the NDM+OXA48-*like* group, susceptibility rates were 18% for colistin and 66.3% for cefiderocol. As anticipated, none of the NDM-producing strains, whether alone or combined with OXA48, were susceptible to ceftazidime/avibactam, consistent with the known ineffectiveness of this combination against metallo-β-lactamases.

In 2024, susceptibility patterns shifted slightly: the OXA48/KPC group showed increased susceptibility to colistin (62%) but a decrease to cefiderocol (58.3%), and 98% to ceftazidime/avibactam (with one KPC strain resistant). The NDM/VIM group exhibited decreased susceptibility to both colistin (41.4%) and cefiderocol (42.3%). The NDM+OXA48-*like* group maintained low colistin susceptibility (18%) but showed improved susceptibility to cefiderocol (73.5%). Consistent with previous findings, no NDM-producing strains, alone or in combination with OXA48, were susceptible to ceftazidime/avibactam.

These observations are presented in the table below (Table 6).

The in vitro activity of meropenem/vaborbactam was assessed against 99 multidrug-resistant (MDR) *K. pneumoniae* isolates, including 84 carbapenemase-producers (KPC, NDM, OXA-48, and NDM+OXA-48-*like*) and 15 ESBL-producers. Susceptibility was determined via disk diffusion on Mueller–Hinton agar with meropenem/vaborbactam (20/10 µg) disks, with results interpreted using EUCAST guidelines.

As anticipated, meropenem/vaborbactam demonstrated no activity against isolates producing metallo-β-lactamases or OXA-48-*like* enzymes. Consequently, 73 of the 99 strains (73.7%) were resistant, a group that included all NDM, OXA-48, and NDM+OXA-48-*like* producers, in addition to 21 KPC-producing isolates. The remaining 26 strains (26.3%) were susceptible, a cohort composed of 11 KPC-producers and all 15 ESBL-producing isolates. The testing of metallo-β-lactamase and OXA-48-*like* producers was performed primarily to confirm their resistance profile for accurate classification within the extensively drug-resistant (XDR) category.

Over the two-year study period, 248 *K. pneumoniae* isolates producing the NDM+OXA48-*like* carbapenemase combination were identified. Among these, 51 isolates (20.5%) demonstrated pan-resistance to all tested antibiotics across multiple classes, including colistin (MIC > 2 mg/L) and cefiderocol (MIC > 2 mg/L). Antimicrobial susceptibility testing was performed using broth microdilution (Micronaut/Bruker), with interpretation based on EUCAST 2023–2024 criteria. These 51 isolates were recovered from diverse clinical specimens: urine (20), lower respiratory tract secretions (15), blood (8), purulent exudates (6), and other sources (2).

To evaluate potential therapeutic options against these extensively drug-resistant strains, the E-test cross method was employed to assess synergy between ceftazidime/avibactam and aztreonam. This method is pertinent for strains producing β-lactamases from Ambler classes A, C, and D, alongside class B metallo-β-lactamases (NDM). Avibactam effectively inhibits serine β-lactamases (classes A, C, D), thereby restoring aztreonam activity against metallo-β-lactamases. This antibiotic combination is increasingly considered for clinical management of infections caused by such multidrug-resistant organisms.

**Figure 6:**
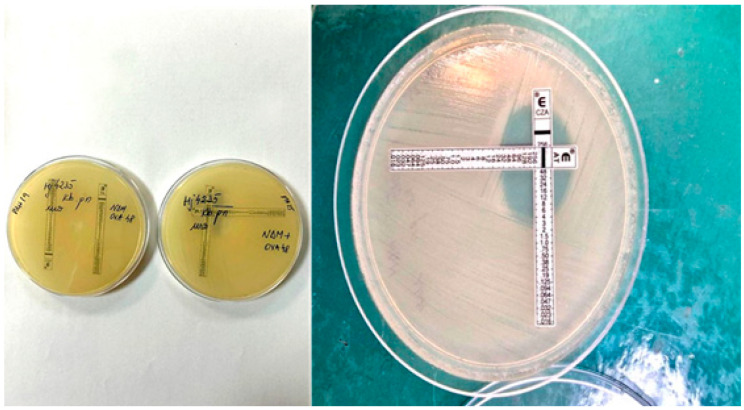
“Perpendicular MIC Test Strip”—synergistic effect of ceftazidime/avibactam + aztreonam. Source: VBH Image Collection (2024).

Synergistic activity was observed across all 51 colistin- and cefiderocol-resistant OXA48 + NDM-producing isolates, with fractional inhibitory concentration (FIC) indices ≤ 0.5, confirming potent synergy (Figure 6).

## 4. Discussion

This study, conducted in a tertiary care centre specialising in infectious diseases, revealed a steady increase in infections caused by CRKP from 2010 to 2024. The prevalence of CRKP rose from 4.9% in 2010 to 40% in 2019, remaining relatively stable during the COVID-19 pandemic, with a moderate increase observed in 2023 and 2024 (45.8% and 41.6%, respectively).

While the overall proportion of CRKP among *K. pneumoniae* isolates remained relatively stable over the last six years, a significant shift occurred in the carbapenemase types. Until 2020, OXA48 predominated; however, following the emergence of strains harbouring combined carbapenemases (NDM+OXA48-*like*) in 2021, these dual producers have become the dominant group by 2023–2024, displacing OXA48 and KPC types. During the pandemic, diagnostic challenges led to a decline in the total number of detected CRKP isolates, but numbers rebounded post-pandemic, exceeding 2018 levels. This increase was more pronounced during the period 2022–2023.

The initial phase of the COVID-19 pandemic saw a marked increase in antibiotic use, particularly carbapenems (meropenem) and third-generation cephalosporins (ceftriaxone), a trend mirrored globally [37,38]. Antibiotic use later stabilised but remained elevated compared to pre-pandemic levels. Notably, colistin consumption rose steadily from 2019 to 2024, likely reflecting an increase in Gram-negative infections, culminating in a nearly 186% increase in 2024 compared to 2019.

These findings, from a tertiary infectious disease hospital with an active antimicrobial stewardship program, suggest that similar or greater antibiotic consumption patterns may have occurred in many general hospitals across Romania. This points to a probable causal link between increased use of injectable β-lactams and colistin and the rise in CRKP strains producing NDM+OXA48-*like* carbapenemases involved in infectious diseases.

In 2023, carbapenem resistance was attributed to 190 carbapenemase-producing CRKP strains: 21 KPC, 34 OXA48, 18 NDM, 112 NDM+OXA48-*like*, and five NDM + KPC. Notably, the most prevalent carbapenemase combination was NDM+OXA48-*like*, representing 58% of all tested CRKP strains. Within this group, 18% of isolates were susceptible to colistin and 66.3% to cefiderocol.

In 2024, 217 carbapenemase-producing CRKP strains (41.4% prevalence) were isolated: 20 KPC, 30 OXA48, 29 NDM, 136 NDM+OXA48-*like*, and one NDM+KPC. The NDM+OXA48-*like* group showed 18% susceptibility to colistin and 73.5% to cefiderocol.

Between 2023 and 2024, the proportion of CRKP strains producing NDM+OXA48-*like* carbapenemase combination increased from 58.9% to 62.7%. In 2024, the number of NDM and OXA48 producers were nearly equal (29 and 30 isolates, respectively), with both exceeding the 18 NDM isolates identified in 2023.

Across the combined 248 NDM+OXA48-*like* isolates from both years, susceptibility rates were 16.5% for colistin, 58.5% for cefiderocol, and 2.8% for amikacin. Notably, 51 isolates (20.5%) exhibited resistance to all tested antibiotics across multiple classes, including colistin and cefiderocol. Using the E-test cross method to assess cumulative susceptibility to ceftazidime/avibactam and aztreonam, we demonstrated synergy between these antibiotics in all colistin- and cefiderocol-resistant NDM+OXA48-*like* strains.

Phenotypic analysis of antibiotic resistance has led to the conclusion that these CRKP isolates, characterised by double or triple associations of different carbapenemases (including metallo-β-lactamases/NDM), pose a significant challenge for clinicians regarding the drastic limitation of therapeutic options. In addition to producing carbapenemases, these strains harbour various resistance mechanisms, both against β-lactams and other classes of antibiotics, which categorise their resistance phenotypes as XDR, possibly even PanDR.

In certain situations, in agreement with the clinician, the CRKP strains were tested against other new antibiotics, such as meropenem/vaborbactam, imipenem/relebactam, tigecycline, and eravacycline, considering the type of biological sample (infection localisation) and the EUCAST interpretation guidelines.

In the meropenem/vaborbactam formulation, vaborbactam is a boronic acid inhibitor that reversibly binds to meropenem. The combination provides stability of meropenem against Class A (KPC) and C β-lactamases but is inactivated by enzymes from Classes B (NDM/VIM) and D (OXA-48) [39]. The CRKP strains producing KPC were tested using the standard Kirby–Bauer disc diffusion method, and all were sensitive to this combination.

Tigecycline is indicated for skin and soft tissue infections, complicated intra-abdominal infections, and community-acquired pneumonia, in patients over 18 years old. It should be considered as a last treatment option for infections caused by CPKp isolates from patients with ventilator-associated pneumonia or systemic infections. If it were the sole treatment option, high doses would be required, as it is bacteriostatic. In recent years, studies have demonstrated a link between increased mortality rates and its improper use [40]. During 2023–2024, the rise in the incidence of NDM+OXA48-*like* carbapenemase-producing CRKP strains resistant to colistin and cefiderocol prompted testing with tigecycline for patients with aspiration pneumonia and systemic infections. MIC values (E-test, BioMerieux) ranged between ≤0.5–1 mg/L, without an interpretation according to EUCAST guidelines for this species, but with a possible clinical efficacy recommendation.

Ceftazidime/avibactam is the recommended combination as the first-line treatment option for complicated intra-abdominal infections, complicated urinary tract infections, or ventilator-associated pneumonia. The avibactam component has a broad spectrum and inhibits enzymes from Ambler classes A, C, and some β-lactamases from class D. This combination is recommended for difficult-to-treat infections, except for infections caused by *K. pneumoniae* producing MBL/NDM enzymes, against which it is not active [41]. The combination with aztreonam has been demonstrated to increase aztreonam’s efficacy in treating infections caused by NDM-producing CRKP strains through a synergistic effect, observed in all 51 NDM+OXA48-*like* strains resistant to both colistin and cefiderocol.

Cefiderocol is stable against serine enzymes (class A/KPC), carbapenemases from classes B (MBL/NDM) and D (OXA), as well as other resistance mechanisms such as porin channel modifications and efflux pump overexpression. It binds to iron ions and is actively transported into the bacterial cell, ensuring increased concentration of the antibiotic at the site of action. Through bacterial siderophores, it acts as a “Trojan horse,” exerting a bactericidal effect [42]. In 2023–2024, the sensitivity of NDM+OXA48-*like* producing CRKP strains to cefiderocol was only 58.5%, mainly due to the combined presence of these two types of carbapenemases.

60% of the biological samples analysed during 2023–2024, from which CRKP strains were isolated, were urine samples. Out of the 51 *K. pneumoniae* strains producing NDM + OXA-48-*like* enzymes and resistant to colistin and cefiderocol, 20 were isolated from urine cultures. Therefore, complicated urinary tract infections posed the most significant therapeutic challenges, representing difficult decision-making scenarios for the antibiotic stewardship team. New antibiotics administered either as monotherapy or in combination included ceftazidime/avibactam with aztreonam and cefiderocol. However, decision-making was limited by restrictions related to the lack of adequate urinary concentrations of certain new antibiotics and by the unavailability of formulations such as aztreonam/avibactam.

The emergence and rapid spread of these XDR strains were linked to the reintroduction of colistin into anti-infective therapy in the mid-1990s [43]. Increased use of colistin, along with improper administration, has led to modifications in the lipopolysaccharide (LPS) structure of Gram-negative bacteria. These changes result from mutations in the pmrCAB and arnBCADTEF-pmr operons, associated with increased positive charge in the LPS and a secondary reduction in colistin affinity [44]. The increased use of colistin, whether as monotherapy or in combination (especially with carbapenems), as observed in our study, has contributed to the rise in colistin resistance, which primarily leads to increased morbidity and mortality [45,46,47].

This study aimed to evaluate the evolution of bacterial strains whose prevalence has accelerated in recent years in hospitals worldwide. Our medical center primarily treats patients with severe infections or multidrug-resistant organisms; therefore, we acknowledge that our findings may not be directly generalizable to those observed in general hospitals across Romania. Nevertheless, the results of this work serve as a significant warning regarding the consequences of recent and current management of bacterial infections, which exert a positive selection pressure favouring the emergence of extensively drug-resistant (XDR) and pandrug-resistant (PDR) strains of *K. pneumoniae*.

As this is a retrospective study, we recognise that missing data may have interfered with the conclusions drawn. The authors also acknowledge that a comprehensive evaluation of the antimicrobial resistance of all identified CRKP isolates was not possible due to various technical constraints encountered during the study period. Similarly, we were unable to assess all potential therapeutic options, such as eravacycline, an antibiotic described in the literature as active against certain MDR Gram-negative infections [48].

## 5. Conclusions

The increasing number of *K. pneumoniae* strains producing carbapenemases, especially those with combined mechanisms such as NDM + OXA-48-*like* enzymes, poses a significant threat to public health and requires active involvement from healthcare systems. Practical strategies to reduce the incidence of these infections should focus on conserving “reserve” antibiotics through the implementation of robust antibiotic stewardship programs across all healthcare facilities, regardless of their profile. Strict adherence to hygiene protocols remains essential in care centers for chronically ill and elderly patients.

To strengthen this “rapid response” capacity, it is crucial to enhance microbiology laboratories’ ability to identify complex resistance mechanisms, incorporate genetic sequencing techniques for bacterial genome analysis to detect circulating resistance genotypes worldwide, and promote active information exchange between laboratories.

## Figures and Tables

**Table 1:** Evolution of Carbapenem-Resistant *K. pneumoniae* Strains (2018–2024).

Year	Total Strains Isolated	Classified Strains	Most Common Carbapenemases/Combinations	Remarks
2018	134	117	OXA48: 85, KPC: 15, NDM: 14	OXA-48 was most prevalent
2019	71	39	OXA48: 30, NDM: 8	OXA-48 most frequent
2020	60	41	OXA48: 30, KPC: 8	Predominance of OXA-48
2021	46	43	OXA48-*like* + NDM: 19, KPC: 18, OXA48: 5, NDM+OXA48-*like* + KPC: 1	First detection of multiple carbapenemase genes
2022	100	62	OXA48-*like* + NDM: 41.93%	Most common co-occurrence
2023	190	190	OXA48-*like* + NDM: 58.9%	Dominant double carbapenemase producers
2024	217	217	OXA48-*like* + NDM: 62.7%	Most frequent co-occurrence

**Table 2:** Resistance to the main antibiotics of *K. pneumoniae* strains isolated in 2023–2024.

Antibiotic Class	2023 Resistance (%) N = 406 Isolates	2024 Resistance (%) N = 521 Isolates	Variation (%)
Amoxicillin–clavulanate	67	63	−4.00
Piperacillin–tazobactam	59.1	62.6	3.50
Ceftriaxone	66.7	63.8	−2.90
Ceftazidime	64	61.2	−2.80
Gentamicine	50	47.5	−2.50
Amikacin	43.3	42.4	−0.90
Ciprofloxacin	66.7	62.5	−4.20
Meropenem	44.6	41.4	−3.20
Imipenem	44.6	41.4	−3.20

**Table 3:** Distribution of carbapenemase types encountered during the period 2023–2024.

Carbapenemase Type	2023 (%)	2023 No. of Strains	2024 (%)	2024 No. of Strains	Change (Percentage Points)
NDM+OXA48-*like*	58.00	110	62.68	136	4.68
OXA48	18.30	35	13.82	30	−4.48
NDM	9.70	18	13.36	29	3.66
KPC	11.30	21	9.22	20	−2.08
NDM + KPC	2.70	5	0.46	1	−2.24
NDM + KPC + OXA48	0	0	0.46	1	0.46

**Table 4:** Antibiotic sensitivity of carbapenem-susceptible OXA48-producing *K. pneumoniae* strains.

OXA48 Strains 2023	IPM	MEM	ETP	AK	GN	SXT	CZA	MIC mg/L CO
1	I	I	I	S	R	R	S	16 (R)
2	S	S	R	S	R	S	S	0.25 (S)
3	S	S	R	S	S	R	S	16 (R)
4	S	S	R	S	R	R	S	4 (R)
5	S	S	R	S	S	R	S	0.25 (S)
6	S	S	R	S	S	R	S	16 (R)
7	S	S	R	S	R	R	S	-

Legend: AK/amikacin, IPM/imipenem, ETP/ertapenem, GN/gentamicin, MEM/meropenem, SXT/trimethoprim/sulfamethoxazole, CZA/ceftazidime/avibactam, CO/colistin.

**Table 5:** Antibiotic susceptibility of 17 out of 30 *K. pneumoniae* strains producing OXA48 carbapenemase.

OXA48 Strains 2024	AK	GN	ATM	CRO	CAZ	MEM	SXT	CZA	CO	FDC
1	S	S	R	R	R	S	R	S	S	S
2	S	S	S	S	S	I	S	S	R	S
3	S	R	R	R	R	S	R	S	S	S
4	S	R	R	R	R	I	R	S	S	R
5	S	S	R	R	R	R	S	S	S	S
6	S	R	R	R	R	R	R	S	S	S
7	S	R	R	R	R	S	R	S	R	S
8	S	S	R	R	R	S	R	S	R	R
9	R	R	I	S	S	I	S	S	S	S
10	S	S	S	R	S	R	R	S	S	S
11	R	R	S	I	S	R	R	S	S	S
12	S	R	R	R	R	R	R	S	S	R
13	R	R	S	R	S	R	R	S	S	S
14	R	R	S	I	S	R	R	S	S	S
15	R	R	I	I	I	R	R	S	S	S
16	R	R	S	I	S	I	R	S	R	S
17	R	R	S	I	S	R	R	S	R	S

Legend: AK/amikacin, GN/gentamicin, ATM/aztreonam, CRO/ceftriaxone, CAZ/ceftazidime, MEM/meropenem, SXT/trimethoprim/sulfamethoxazole, CZA/ceftazidime/avibactam, CO/colistin, FDC/cefiderocol.

**Table 6:** Antibiotic susceptibility of “old” and “new” molecules by carbapenemase types (2023, 2024).

Carbapenemase	Year	Colistin (%)	Cefiderocol (%)	Ceftazidime/Avibactam (%)
OXA48/KPC	2023	48	75.6	100
	2024	62	58.3	98
Variability		14	−17.3	−2
NDM/VIM	2023	50	58.3	0
	2024	41.4	42.3	0
Variability		−8.6	−16	0
NDM+OXA48-*like*	2023	18	66.3	0
	2024	18	73.5	0
Variability		0	7.2	0

## Data Availability

The corresponding author can provide the data used in this study upon reasonable request.

## Abbreviations

The following abbreviations are used in this manuscript:

AVI	Avibactam
BMD	Broth microdilution method
CAMHB	Mueller–Hinton Broth, cation-adjusted
CAZ	Ceftazidime
CFIII	Third-generation cephalosporins
CPE	Carbapenemase-producing Enterobacteriaceae
CRE	Carbapenem-resistant Enterobacteriaceae
CRKP	*K. pneumoniae* carbapenem-resistant
ESBL	Extended-spectrum β-lactamase
FIC	Fractional inhibitory concentration
KPC	*K. pneumoniae* carbapenemase
MALDI-TOF	Matrix-Assisted Laser Desorption/Ionization—Time of Flight Mass Spectrometry
MBL	Metallo-β-lactamase
MDR	Multidrug-resistant
MIC	Minimum inhibitory concentration
NDM	New Delhi metallo-β-lactamase
OXA-48	Oxacillinase β-lactamase-48
PDR	Pandrug-resistant
TU	Therapeutic units
VBH	“Dr. Victor Babeș” Clinical Infectious and Tropical Diseases Hospital in Bucharest
VIM	Verona integron-encoded metallo-β-lactamase
XDR	Extensively drug-resistant

## Appendix A. The Methodology of Broth Microdilution

MICRONAUT-S (Bruker) are microtitration plates for automated susceptibility testing of clinically aerobic Gram-negative bacteria. MICRONAUT-S test plates are in vitro diagnostic medical devices for qualitative phenotypic detection and differentiation of relevant carbapenemases and cephalosporinases in clinically relevant, fast-growing, aerobic Gram-negative bacteria. Susceptibility to antibiotics is detected by determining MIC concentrations according to EUCAST and CLSI. Only pure cultures can be used.

The susceptibility testing is based on the rehydration of antibiotics by adding a standardized bacterial suspension. On a tube with 2–5 mL NaCl 0.9%, Ph 5.5 to 6.5, add the colonies until the turbidity matches a McFarland standard of 0.5. After that, pipette 50 µL of the bacterial suspension into 11.5 mL CAMBH (Mueller Hinton Broth cation-adjusted) and homogenize well. Inoculate the microtitration plate with 100 µL per well. After incubation for 18–24 h at 35–37° Celsius, the result is read with the plate reader and evaluated with the MICRONAUT software.

UMIC^®^ Cefiderocol (Bruker) are ready-to-use strips with 12 wells, based on the broth microdilution reference method recommended by EUCAST and CLSI. The UMIC Cefiderocol is suitable for determining the MIC values in the range of 0.03 to 32 mg/L for Enterobacterales, Pseudomonas aeruginosa, and Acinetobacter baumannii. On a tube with 2–5 mL NaCl 0.9%, pH 5.5 to 6.5, add the colonies until the turbidity matches a McFarland standard of 0.5. Then pipette 25 µL from the bacterial suspension into the Iron-depleted CAMBH and homogenize. Inoculate the strip with 100 µL per well. After incubation for 18–24 h at 35–37° Celsius in UMIC incubation boxes to preserve a humid atmosphere, the result is read visually.
